# Synergistic Anti-Cancer Effect of Phenformin and Oxamate

**DOI:** 10.1371/journal.pone.0085576

**Published:** 2014-01-21

**Authors:** W. Keith Miskimins, Hyun Joo Ahn, Ji Yeon Kim, Sun Ryu, Yuh-Seog Jung, Joon Young Choi

**Affiliations:** 1 Cancer Biology Research Center, Sanford Research/USD, Sioux Falls, South Dakota, United States of America; 2 Department of Obstetrics and Gynecology and Division of Basic Biomedical Sciences, Sanford School of Medicine of the University of South Dakota, Sioux Falls, South Dakota, United States of America; 3 Department of Anesthesiology and Pain Medicine, Samsung Medical Center, Sungkyunkwan University School of Medicine, Seoul, Korea; 4 Head and Neck Oncology Clinic, Center of Specific Organs Cancer, Center for Thyroid Cancer, Research Institute and Hospital, National Cancer Center, Goyang-si, Gyeonggi-do, Korea; 5 Department of Nuclear Medicine, Samsung Medical Center, Sungkyunkwan University School of Medicine, Seoul, Korea; Instituto Nacional de Cardiologia, Mexico

## Abstract

Phenformin (phenethylbiguanide; an anti-diabetic agent) plus oxamate [lactate dehydrogenase (LDH) inhibitor] was tested as a potential anti-cancer therapeutic combination. In *in vitro* studies, phenformin was more potent than metformin, another biguanide, recently recognized to have anti-cancer effects, in promoting cancer cell death in the range of 25 times to 15 million times in various cancer cell lines. The anti-cancer effect of phenformin was related to complex I inhibition in the mitochondria and subsequent overproduction of reactive oxygen species (ROS). Addition of oxamate inhibited LDH activity and lactate production by cells, which is a major side effect of biguanides, and induced more rapid cancer cell death by decreasing ATP production and accelerating ROS production. Phenformin plus oxamate was more effective than phenformin combined with LDH knockdown. In a syngeneic mouse model, phenformin with oxamate increased tumor apoptosis, reduced tumor size and ^18^F-fluorodeoxyglucose (FDG) uptake on positron emission tomography/computed tomography compared to control. We conclude that phenformin is more cytotoxic towards cancer cells than metformin. Furthermore, phenformin and oxamate have synergistic anti-cancer effects through simultaneous inhibition of complex I in the mitochondria and LDH in the cytosol, respectively.

## Introduction

Observations that metformin (1,1-dimethylbiguanide), the most commonly prescribed drug for type II diabetes reduces cancer risk have promoted an enthusiasm for metformin as an anti-cancer therapy [1], [2]. Now clinical trials in breast cancer using metformin alone or in combination with other therapies are underway [3], [4].

Phenformin, another biguanide (1-phenethylbiguanide) was introduced at the same time as metformin, in the late 1950s as an anti-diabetic drug. Phenformin is nearly 50 times as potent as metformin but was also associated with a higher incidence of lactic acidosis, a major side effect of biguanides. Phenformin was withdrawn from clinical use in many countries in the late 1970s when an association with lactic acidosis and several fatal case reports was recognized [5]. Consequently, the effect of phenformin on cancer has rarely been studied.

To prevent the development of resistant cancer cells, rapid and complete killing of cancer cells by chemotherapy is important. It is therefore possible that phenformin can be a better anti-cancer agent than metformin due to its higher potency. In one *in vivo* study, established breast tumors treated with metformin did not show significant inhibition of tumor growth, whereas phenformin demonstrated significant inhibition of tumor growth [6].

The mechanisms by which metformin inhibits cancer development and tumor growth are not completely understood. Suggested mechanisms include activation of AMP-activated protein kinase (AMPK) [7], inhibition of mTOR activity [8], Akt dephosphorylation [9], disruption of UPR transcription [10], and cell cycle arrest [11]. Recently, it was revealed that the anti-diabetic effect of metformin is related to inhibition of complex I in the respiratory chain of mitochondria [12], [13]. However, complex I has never been studied with regard to the anti-cancer effect of biguanides.

Therefore, in this study we aimed to first test whether phenformin has a more potent anti-cancer effect than metformin and if so, investigate the anti-cancer mechanism. We hypothesized that phenformin has a more potent anti-cancer effect than metformin and that its anti-cancer mechanism involves the inhibition of complex I.

In addition, we combined oxamate, a lactate dehydrogenase (LDH) inhibitor, with phenformin to reduce the side-effect of lactic acidosis. Oxamate prevents the conversion of pyruvate to lactate in the cytosol and thus prevents lactic acidosis. Interestingly, lactic acidosis is a common phenomenon in the cancer microenvironment and is related to cancer cell proliferation, metastasis, and inhibition of the immune response against cancer cells [14], [15]. Recent experiments showed that LDH knockdown prevented cancer growth [16], [17], therefore addition of oxamate may not only ameliorate the side effect of phenformin but might also itself inhibit the growth and metastasis of cancer cells.

No studies have tested phenformin in combination with oxamate, either *in vitro* or in immune competent syngeneic mice. In this study, we investigate whether phenformin and oxamate have a synergistic anti-cancer effects by simultaneous inhibition of complex I in the mitochondria and LDH in the cytosol through both *in vitro* tests and in a syngeneic mouse model.

## Materials and Methods

Four groups were compared in this study: control group (group C), phenformin group (group P), oxamate group (group O), and a combination group of phenformin and oxamate (group PO). All measurements in *in vitro* studies were performed 1 day after drug treatment unless otherwise specified.

### Chemicals and Cell Culture

Metformin (1,1-dimethylbiguanide), phenformin (1-phenethylbiguanide), and sodium oxamate were purchased from Sigma Chemicals and were diluted with sterile water to different concentrations. PARP inhibitor (INH2BP, 5-Iodo-6-amino-1,2-benzopyrone) was purchased from Calbiochem and caspase inhibitor (Q-Val-Asp-OPh) was purchased from MP Biomedicals. The cell lines MCF7 (breast cancer), B16F10 (melanoma), CT26 (colon cancer), A549 (lung cancer), and DU145 (prostate cancer) were purchased from American Type Culture Collection (ATCC). The E6E7Ras (tonsil cancer) was obtained from Dr J Lee (Sanford Research, Cancer Biology Research Center) [18], [19]. All cells were maintained in Dulbecco's modified Eagle's medium (DMEM) containing 10% fetal bovine serum and supplemented with 100 U/ml penicillin and 100 µg/ml streptomycin in a humidified incubator with 5% CO_2_. Drugs were administered at a cell confluency of 70%.

### Determination of Drug Dosage

CT26, a colon cancer cell line from BALB/c mice, was chosen as the primary system of study because CT26 cells are relatively resistant to phenformin but showed a dramatic synergistic effect upon the addition of oxamate. Additionally, our syngeneic mouse experiments were performed in BALB/c mice.

MCF10A cells, a non-transformed human mammary epithelial cell line, remained unaffected in the presence of up to 1 mM phenformin plus 40 mM oxamate for 1 week. However, higher doses produced cell death (data not shown). Therefore, we used 1 mM phenformin, 40 mM oxamate, and 1 mM phenformin plus 40 mM oxamate for further experiments.

### Measurement of Cell Death by Trypan Blue Exclusion Assays and Flow Cytometry

Cells were plated in 35 mm dishes and treated with or without drugs. For the trypan blue exclusion assay, a cell suspension was stained with 0.02% trypan blue. Trypan blue positive and negative cells were counted using a hemacytometer. For flow cytometry measurements, 7-aminoactinomycin D (7AAD; 5 µl) was added to 500 µl cell suspension and incubated for 20 minutes on ice. All flow cytometry measurements were performed using a BD Accuri C6 flow cytometer (BD Biosciences).

A dose-response curve, EC_50_, and combination index (CI) was obtained using Calcusyn software (Version 2.1, BIOSOFT).

### Measurement of pH and Lactate

pH of culture media was measured using a pH meter (Accumet AB15 Basic and BioBasic pH/mV/°C meter, Fisher Scientific). Lactate in culture media was measured using a lactate assay kit (Eton Bioscience, Inc.) and microplate reader (absorbance 490 nm, SpectraMax Plus^584^, Molecular Devices) in a quantitative manner with lactate standards. Lactate production was standardized per 10^5^ cells.

### Complex I Activity

Complex I activity was determined from the oxidation rate of NADH (Fluka) per mg protein. Cell pellets were sonicated for 20 sec on ice in IME buffer (50 mM imidazole, 2 mM MgCl_2_, 1 mM EDTA, Protease inhibitors) and 80 µg cell extract was added to reaction buffer [1 mM EDTA, 50 mM KCl, 1 mM KCN, 1.2 µM antimycin A, 10 mM Tris-HCl (pH 7.4)]. Just before measurement, 150 µM NADH and 100 µM coenzyme Q1 (Sigma), as an electron acceptor, were added. Absorbance at 340 nm was measured over 2 minutes using a spectrophotometer at 30°C. NADH oxidation not blocked by rotenone (a complex I inhibitor, 2.5 µM) was removed from the calculation to measure NADH oxidation occurring in complex I only.

To validate a role for complex I inhibition by phenformin, 0.5 mM methyl succinate (Sigma) was added to complete growth media with phenformin at the same time to observe if phenformin’s anti-cancer cell effects were reversed. Methyl succinate serves as an alternate energy source that bypasses complex I in the electron transport chain. Cell death was measured 24 hours after treatment.

### LDH Activity

LDH activity was determined by monitoring the rate of NADH consumption upon addition of pyruvate. Cell pellets were resuspended in 0.1 M KH_2_PO_4_ (pH 7.2), 2 mM EDTA, and 1 mM dithiothreitol (DTT), sonicated in 300 µl assay buffer (50 mmol/L potassium phosphate, pH 7.4), and centrifuged at 10,000 g for 10 minutes at 4°C. The supernatant was added to 50 mM potassium phosphate (pH7.4), 2 mM pyruvate, and 20 µM NADH. Absorbance was measured over 10 minutes using a spectrophotometer at excitation 340 nm and 30°C. LDH activity was standardized per 10^5^ cells.

### Oxygen Consumption Rate (OCR) and Extracellular Acidification Rate (ECAR)

OCR and ECAR were measured using the Seahorse XF24 extracellular flux analyzer (Seahorse Bioscience, Billerica, MA, USA). This device uses a disposable sensor cartridge which is embedded with fluorescence-based optical biosensors (oxygen and protons) that allows for simultaneous extracellular real time measurements of intact cells growing as monolayers. CT26 was seeded at 40,000 cells per well on XF24 V7 multi-well plates and were pre-incubated for 24 h at 37°C in 5% CO_2_. The following day, cells were rinsed with assay media, and then incubated without CO_2_ at 37°C for one hour in assay media (DMEM base, 4 mM glutamine, 143 mM NaCl, 25 mM glucose at a pH of 7.4). After establishing two baseline OCR and ECAR readings, studied drugs were injected and measurements continued for 70 min. After seventy minutes, 10 mM glucose was injected and OCR and ECAR were measured for another 20 min. Experiments were run in quadruplicate.

### Mitochondrial Reactive Oxygen Species (ROS)

Mitochondrial ROS were detected using red mitochondrial superoxide indicator (MitoSOX™, Molecular Probes). MitoSOX™ is a fluorogenic dye for highly selective detection of superoxide in the mitochondria of live cells. Once in the mitochondria, MitoSOX™ Red reagent is oxidised by superoxide and exhibits red fluorescence. Cells grown in a 35-mm glass bottom culture dish (Mat Tak corporation) were incubated with 5 µM MitoSOX™ and 100 nM MitoTracker Green ® (Molecular Probes) for mitochondria staining for 10 minutes at 37°C protected from light. Cells were gently washed three times with warm buffer and mounted in warm buffer for imaging. Olympus FV1000 confocal microscopy was performed at Ex/Em: 510/580 nm.

To validate the importance of ROS production, the ROS scavenger, N acetyl cysteine (NAC, Sigma, 1 mM) was added to complete growth medium 6 hours before test drug administration. Cell death was measured 24 hours after treatment.

### ATP Levels

ATP levels were determined by a luciferin-luciferase-based assay with an ATP Bioluminescence Assay kit (Molecular Probes, Invitrogen). The assay relies on the requirement of luciferase for ATP to produce light. Measurements were obtained using a luminometer (GloMax® 96 Microplate Luminometer, Promega) at an emission maximum of approximately 560 nm for 300 sec. ATP standards were run concurrently with each experiment to produce a standard curve, and calculations were made against the curve to determine cellular ATP levels. ATP was expressed per 10^5^ cells.

### DNA Damage

DNA damage was quantitatively measured by 8-hydroxydeoxyguanosine (8-OHdG) in media, nuclei, and mitochondria. 8-OHdG is a very specific by-product of oxidative damage of DNA and reflects intracellular oxidative stress. Cells were cultured in 35 mm dishes for 8-OHdG detection in media and nuclei, and in 100 mm dishes for mitochondrial 8-OHdG. Nuclei and mitochondria were separated by differential centrifugation. DNA was extracted from nuclei and mitochondria using a commercial DNA extraction kit. DNA was converted to single-stranded DNA by incubation at 95°C for 5 minutes and rapidly chilled on ice. The denatured DNA sample was then digested to nucleosides by incubation with 10 units of nuclease P1 for 2 hrs at 37°C in 20 mM sodium acetate (pH 5.2), followed by treatment with 10 units of alkaline phosphatase for 1 hr at 37°C in 100 mM Tris (pH 7.5). The reaction mixture was centrifuged for 5 minutes at 6,000 g and the supernatant was used for the ELISA 8-OHdG kit (OxiSelect™, Cell Biolabs). The remaining procedure was done following the protocol supplied by the manufacturer of the ELISA 8-OHdG kit. DNA damage was standardized per 10^6^ cells.

### LDH Knock Down

Expression of LDHA was knocked down by siRNA. The target sequence of LDHA was CAACUGCAGGCUUCGAUUA. Thermo Scientific DharmaFECT Transfection Reagents were used according to the manufacturer. Untreated cells and cells transfected with negative control siRNA (non-targeting) or the test siRNA were prepared in triplicate. 165,000 cells were incubated in 35-mm well plates for 1 day and transfected with 15 µl siRNA and 6.8 µl Dharmafect for 2 days. Drug treatment was started after 24 hours of transfection. LDH knockdown was confirmed by western blot analysis after 2 days of transfection (anti-LDHA antibody, 1∶1000, #ab47010, Abcam®).

### Cancer Cell Death

Western blotting and confocal microscopy were performed to detect cleaved PARP [poly (ADP-ribose) polymerase] and apoptosis inducing factor (AIF). PARP is a substrate for caspases and cleaved PARP (cPARP) is a hallmark of caspase-dependent apoptosis. AIF is a hallmark of PARP-dependent cell death. We also used caspase inhibitor and PARP inhibitor to test whether these inhibitors block cancer cell death.

#### Western blotting

Antibodies to PARP (#9542, used at 1∶1000), and AIF (#4642, used at 1∶1000) were purchased from Cell Signaling Technology. cPARP was detected in whole cell lysates and AIF was detected in nuclear extracts. To obtain nuclei for measurement of AIF, cells were washed in cold PBS and suspended in 400 µl ice-cold hypotonic buffer [10 mM HEPES/KOH (pH 7.9), 2 mM MgCl_2_, 0.1 mM EDTA, 10 mM KCL, 1 mM DTT, 0.5 mM PMSF (phenylmethylsulphonyl fluoride) and 1% (v/v) eukaryotic protease inhibitor cocktail] for 10 minutes on ice. The cell pellet was gently resuspended in 100 µl ice-cold saline buffer (50 mM HEPES/KOH (pH 7.9), 50 mM KCl, 300 mM NaCl, 0.1 mM EDTA, 10% glycerol, 1 mM DTT, 0.5 mM PMSF, 1% (v/v) eukaryotic protease inhibitor cocktail) and incubated on ice for 20 minutes. The cell suspension was vortexed and centrifuged at 15,000 g for 5 minutes at 4°C. The supernatant was taken as the nuclear lysate and subjected to SDS polyacrylamide gel electrophoresis (PAGE) and western blot analysis to measure AIF.

#### Confocal microscopy

Cells were washed in PBS and fixed in 4% paraformaldehyde for 15 minutes. For detection of endogenous proteins by immunofluorescence, cells were permeabilized in 0.25% Triton X-100 for 5 minutes and then washed in PBS three times. This was followed by blocking in 10% bovine serum albumin (BSA) in PBS for 30 minutes and incubation in primary antibody for 2 hrs at 37°C. Primary antibody (1∶100) was prepared in 3% BSA in PBS. Slides were washed three times in PBS and incubated with Alexa Fluor 594-labeled secondary antibody (1∶200, Molecular Probes) for 45 minutes. Finally, slides were washed in PBS three times and mounted using Vectashield medium containing 4, 6-diamidino-2-phenylindole (DAPI) (Vector Laboratories). Slides were observed using an Olympus FV1000 confocal microscope.

#### Inhibitor treatment

CT26 cells were pretreated with 1 µM caspase inhibitor (Q-Val-Asp-OPh, MP Biomedicals) or PARP inhibitor (INH2BP, 5-Iodo-6-amino-1,2-benzopyrone, Calbiochem) for 4 hours before treatment with phenformin or phenformin plus oxamate. The percentage of dead cells was counted 24 hours after treatment in the group P and 12 hours after treatment in the group PO by flow cytometry using 7-AAD.

### Mice

Seven week old BALB/c mice (Orientbio Inc. Korea) were used. Experiments were approved by the Institutional Animal Care and Use Committee of Samsung Biomedical Research Institute and were performed in accordance with the ARRIVE (Animals in Research: Reporting In VIVO Experiments) guidelines [20]. All mice were maintained in a pathogen-free animal facility.

#### Treatment regimen

BALB/c mice received saline (Group C, n = 24), oxamate 300 mg/kg (Group O, n = 31), phenformin 17 mg/kg (Group P, n = 31), or phenformin 17 mg/kg +300 mg/kg oxamate (group PO, n = 31). Mice were subcutaneously inoculated with 1×10^7^ CT26 cells in 0.2 ml of PBS on the left flank. Designated drugs of each group were administered intraperitoneally 3 days after cell injection. All drugs were injected in a total volume of 0.25 ml diluted with sterile water. Animals were treated every day for 21 days. Body weight and tumor size were measured 3 times a week. Tumor size was measured with external calipers (Mitutoyo, Japan). Tumor size was estimated using a formula = (d1×d2^2^)/2 in which d1 and d2 are the longest and the shortest diameters of the tumor, respectively, measured in mm.

On day 21 after treatment, mice were anesthetized with 2.5% enflurane in O_2_ and tumors were removed and cut in half. One half of each tumor was snap frozen and the other half fixed in 4% paraformaldehyde in 0.1 M phosphate buffer overnight at 4°C.

#### Apoptosis assay

Tumor tissues were sectioned at a thickness of 10 µm using a cryostat, thaw mounted on gelatin-coated slides and stored at −20°C. To detect apoptosis, tissue sections were stained with the terminal deoxynucleotidyltransferase-mediated dUTP nick-end labeling (TUNEL) method using the Fluorescein *in situ* cell death detection kit (DeadEnd™ Fluorometric TUNEL System; Promega). Slides were observed under a confocal microscope LSM700 (Zeiss, Germany). The FITC-labeled cells undergoing apoptosis were recognized by nuclei with strong green fluorescence. For the quantification, TUNEL positive cells were counted in three sections (304 µm×304 µm) at ×20.

#### ^18^F-FDG small animal PET/CT

PET/CT was performed 24 days after CT26 injection and 21 days after initiating drug treatments. A dedicated small animal PET/CT scanner (Inveon Multimodality System, Siemens Healthcare, Knoxville, TN, USA) was used for the mouse imaging. Its intrinsic spatial resolution and axial field-of-view were 1.4 mm and 12.5 cm, respectively. At first, mice were anesthetized with isoflurane. After CT scan for attenuation correction (tube voltage 60 kVp, tube current 400 µA) was performed, 7.4±3 MBq of ^18^F-FDG was injected via tail vein. PET emission scan for 5 min was performed 60 min after the injection of ^18^F-FDG. One mouse at a time was imaged and kept on a warm pallet during the imaging procedure. After data acquisition, transverse PET images were reconstructed with an ordered subset expectation maximization 3D algorithm (4 iterations) with a voxel size of 0.776×0.776×0.796 mm. CT images were reconstructed using a filtered back projection algorithm with a Shepp–Logan filter.

PET, CT and fused PET/CT images were displayed and analyzed with the Inveon Research Workplace software (Siemens Healthcare). A volume-of-interest (VOI) covering entire tumors were defined based on CT images. Average standardized uptake value (SUV_avg_) of the tumor was obtained by using the VOI from the CT image. SUV was corrected for injected dose of ^18^F-FDG, mouse body weight and tumor size. SUVavg data are displayed as a percentage of baseline in order to easily assess relative changes.

### Statistical Analysis

Statistical analysis was performed with the software program IBM SPSS statistics (SPSS Inc., Chicago, USA). Statistical differences between means were determined by the *t-*test or one-way ANOVA followed by Tukey’s HSD test. Nominal categorical data were compared by Pearson’s chi square. Statistical significance was accepted for p values of <0.05.

## Results

### Phenformin Exhibits Higher Cancer Cell Cytotoxicity than Metformin

**Figure 1:**
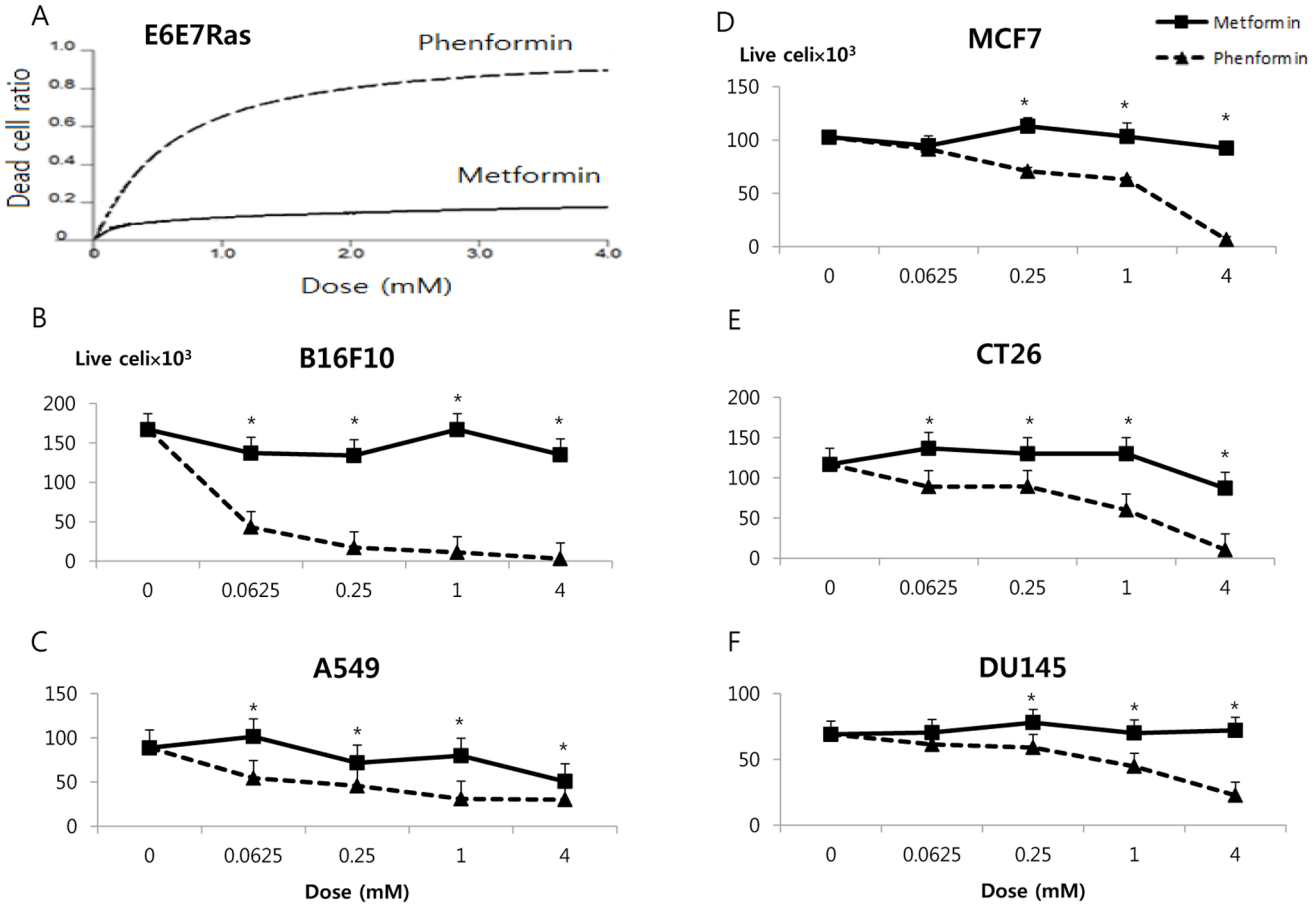
Comparison of dose dependent effects of phenformin and metformin in cancer cell lines. Cells were treated for 2(A) or the number of live cells (B–F) was determined. (A) E6E7Ras cells, a mouse model of HPV+ head and neck squamous cell carcinoma, (B) B16F10 mouse melanoma cells, (C) A549 human lung adenocarcinoma cells, (D) MCF7 human breast cancer cells, (E) CT26 mouse colon cancer cells, and (F) DU145 human prostate cancer cells. *: P<0.05.

Most available data relating to the effects of biguanides on cancer cells, and our own previous work [21]–[23], have concerned metformin. We have previously observed metformin cytotoxicity to MCF7 cells, but this required higher doses over a longer time period [21], [22]. Because of the high levels of metformin required and the possible higher potency of phenformin [24], we wanted to directly compare the cytotoxicity of the two drugs in multiple cancer cell lines. In E6E7Ras cells, a model of HPV+ head and neck squamous cell carcinoma [18], [19], the EC_50_ for metformin and phenformin for promoting cancer cell death were 504 mM and 0.6 mM, respectively. The EC_50_ of metformin was 840 times higher than that of phenformin (Fig. 1A). Phenformin showed excellent cytotoxicity on various other cancer cell lines, where metformin showed little, if any, effect under these conditions (Fig. 1B–F). The EC_50_ of metformin were 15,200,000 times, 448 times, 67 times, 26 times, and 25 times higher than phenformin in B16F10 (melanoma), MCF7 (breast cancer), CT26 (colon cancer), A549 (lung cancer), and DU145 (prostate cancer), respectively.

### Phenformin and Oxamate Exhibited a Synergistic Effect on Cancer Cell Cytotoxicity

**Figure 2:**
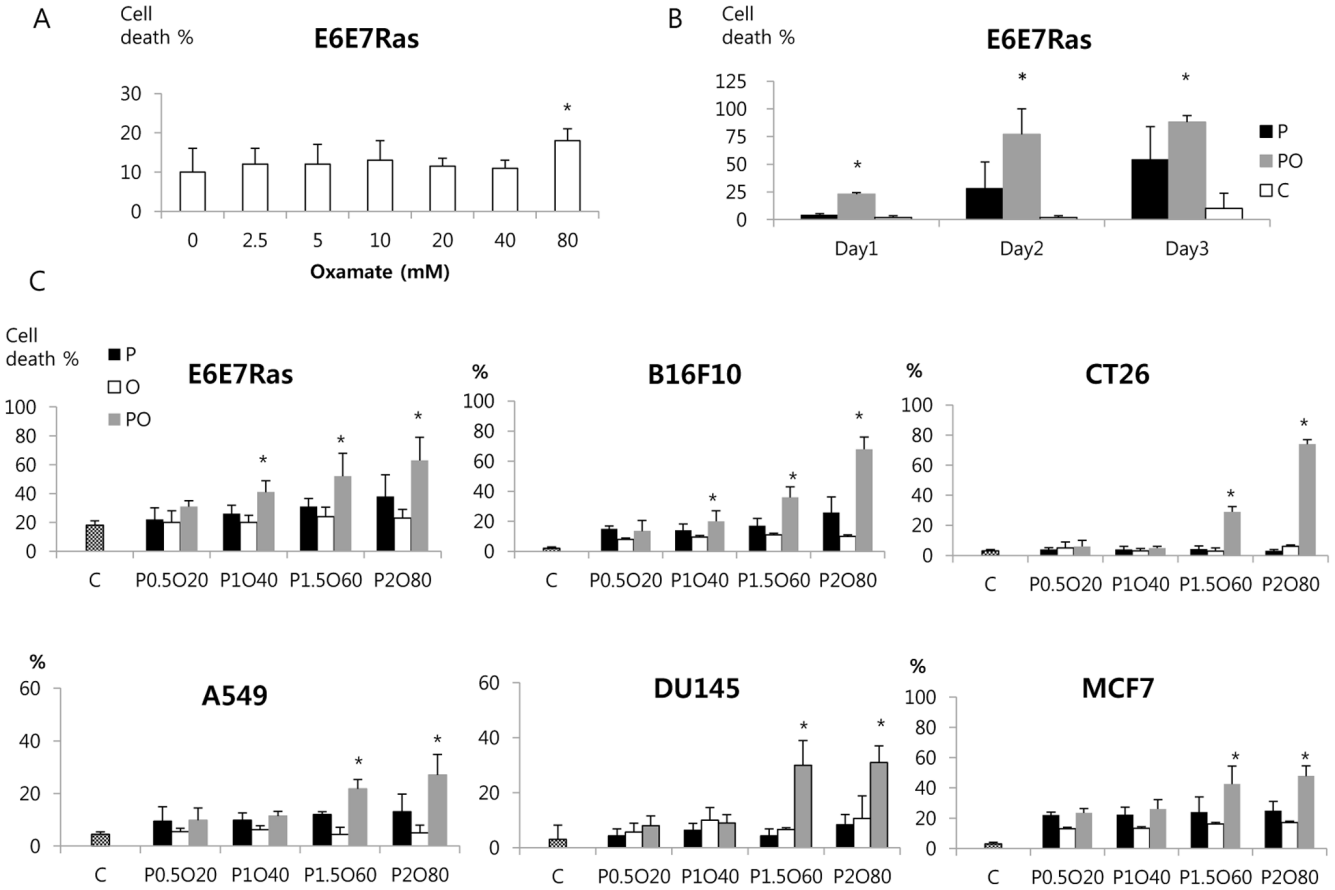
Synergism between phenformin and oxamate in mediating cancer cell death. (A) E6E7Ras cells were treated for 2 days with oxamate at the indicated concentrations (0–80 mM) and then dead cells were counted by flow cytometry. (B, C) The indicated cells lines were treated with varying concentrations of phenformin, oxamate, or combinations of the two drugs. In (B) cells were treated for 1, 2, or 3 days prior to counting dead cells. In (C) cells were treated for 24 hours before determining number of dead cells. C: control, P: phenformin, O: oxamate, PO: phenformin+oxamate. In (C) the numbers below each bar indicate concentrations of each drug in mM (e.g., P0.5O20 means P 0.5 mM+O 20 mM). * indicates a synergistic effect in the group PO compared with the other groups.

Biguanides, e.g. metformin and phenformin, are known inhibitors of complex I of the mitochondrial electron transport chain and our previous studies showed that mitochondria are important targets of metformin in breast cancer cells [22]. Inhibition of mitochondrial metabolism promotes glycolytic metabolism and lactate production and export. We therefore reasoned that inhibiting the conversion of pyruvate to lactate would promote entry of pyruvate into mitochondrial metabolism and enhance the cytotoxic effects of phenformin. Oxamate is a known inhibitor of LDH [25]. In studies presented here, oxamate alone showed a weak cytotoxic effect in the range from 0–80 mM (Fig. 2A). Phenformin alone showed cytotoxic effects but the potency was different between various cancer cell lines (Fig. 2B, 2C). Phenformin and oxamate co-administration however, exhibited a strong synergistic effect on cancer cell killing in all cancer cell lines tested. Combination index (CI) was calculated using the multiple drug-effect equation of Chou-Talalay [26] in the Calcusyn program. CIs reflect the type of interaction between co-administered drugs. CI values in the range 0.9 and 1.1 indicate an additive effect, whereas CI values of <0.9 indicate synergism and CI values of >1.1 indicate antagonism. The combination index (CI) was 0.494 in E6E7Ras, 0.310 in B16F10, 0.009 in CT26, 0.227 in A549, and 0.067 in DU145, and 0.503 in MCF7 (strong synergism) when co-administered as compared with a single administration at ED_50_. Longer treatment (Fig. 2B) and higher doses (Fig. 2C) resulted in increased cytotoxicity in phenformin.

### Effects of Phenformin and Oxamate on Lactate Production and pH

**Figure 3:**
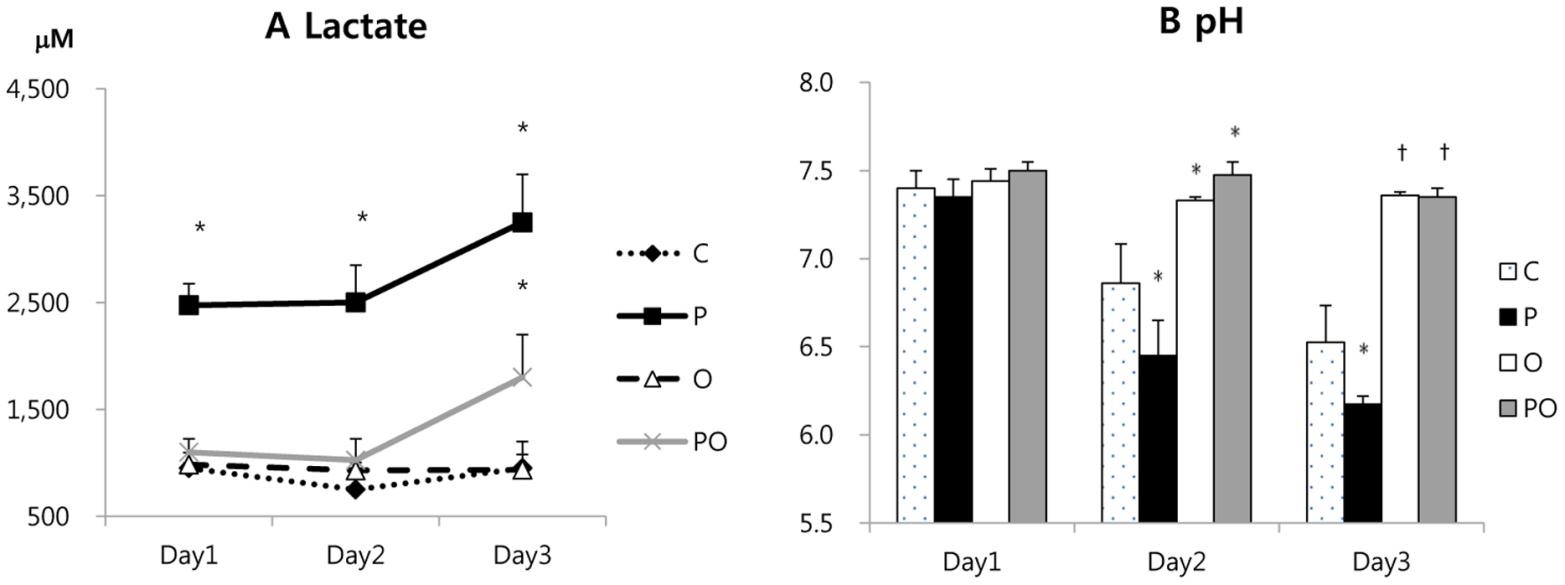
Changes in lactate and pH of the medium in cells treated with phenformin and oxamate. CT26 cells were treated with the indicated compounds for 1, 2, or 3(A) or medium pH (B) was determined. P: phenformin 1 mM, O: oxamate 40 mM, PO: phenformin 1 mM+oxamate 40 mM, C: untreated control. *: P<0.05 compared with the other groups. †: P<0.05 compared with the group C and P.

Biguanides are known to enhance glucose uptake, glycolytic metabolism, and lactate secretion. Oxamate, on the other hand, is an inhibitor of LDH and expected to reduce lactate production by the cells. To examine whether these compounds were affecting the presumed cellular targets, lactate in the culture medium was measured in CT26. Since lactate is transported from the cell together with a proton, medium pH was also measured. Phenformin increased lactate production and decreased medium pH compared with the control, indicating elevated rates of glycolysis. Oxamate decreased lactate production and increased pH, suggesting the expecting inhibition of LDH. Addition of oxamate to phenformin reversed both the increase in lactate production and the decrease in pH caused by phenformin treatment (Fig. 3A, 3B).

### Cytotoxic Effects of Phenformin and Oxamate are Related to Complex I and LDH Inhibition, Respectively

**Figure 4:**
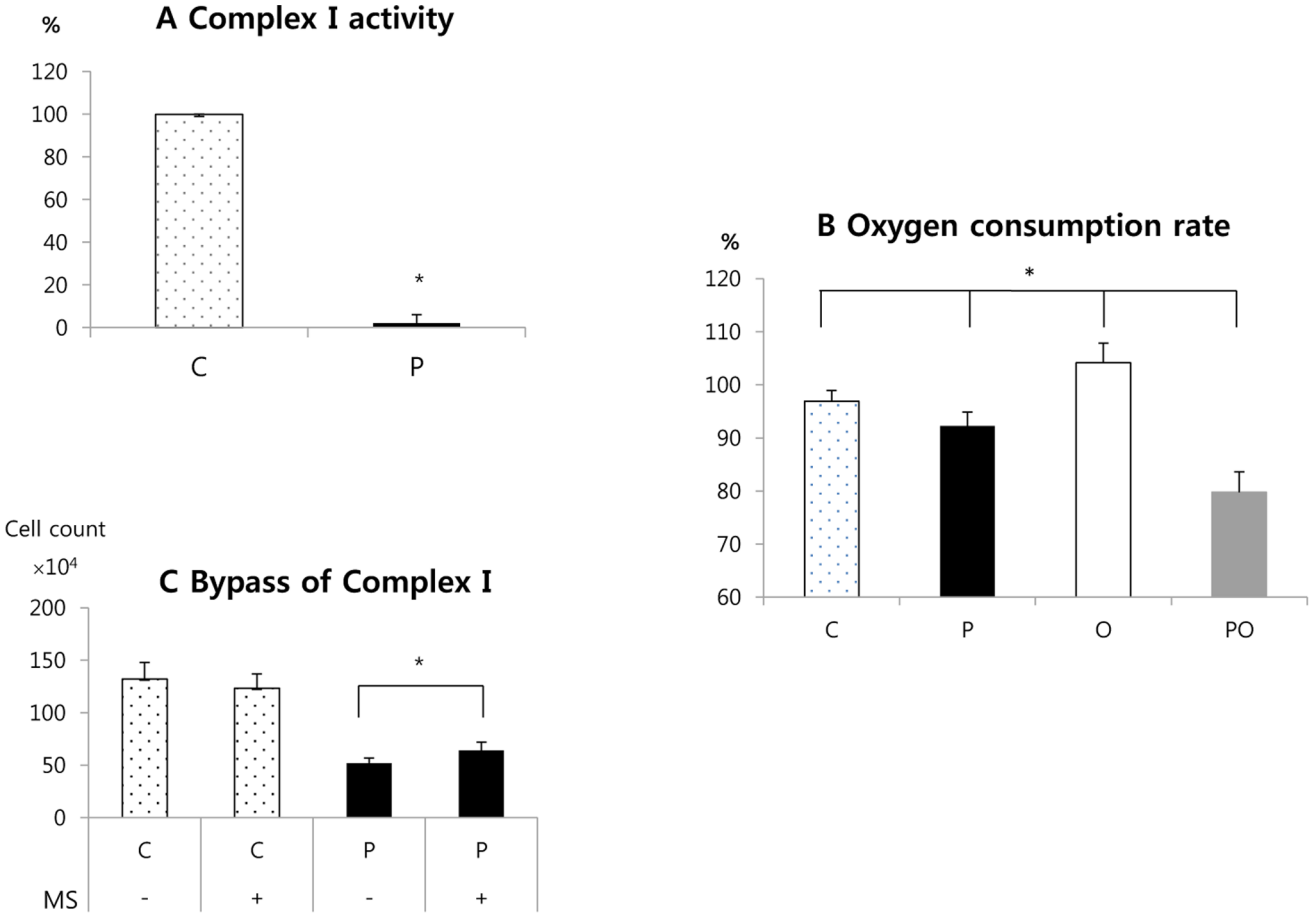
Complex I inhibition by phenformin. (A) CT26 cells were treated with or without phenformin for 24 hours and then extracts were prepared to measure complex I activity as described in Materials and Methods. The Y axis is % of complex I activity when the activity of complex I in the control group is regarded as 100%. (B) Effects of the indicated compounds on oxygen consumption by CT26 cells were determined as an indicator of mitochondrial oxidative metabolism. (C) Cells were treated with or without phenformin in the presence or absence of methyl succinate, which bypasses complex I of the electron transport chain. After 24 hours the number of live cells in the cultures was determined. MS: methyl succinate. C: control, P: phenformin 1 mM, O: oxamate 40 mM, PO: phenformin 1 mM+oxamate 40 Mm. *: P<0.05.

As described above, the putative targets of phenformin and oxamate are complex I of the mitochondrial electron transport chain and LDH, respectively. The changes in lactate in response to these compounds support this conclusion. The following experiments were designed to more directly define the effects of the compounds on their putative targets. First, the effects of phenformin on complex I activity was directly measured as described in Materials and Methods. Phenformin treatment of cells strongly inhibited mitochondrial complex I activity (Fig. 4A). To further substantiate this finding, mitochondrial oxidative metabolism was measured by the Seahorse XF24-3 extracellular flux analyzer following treatment of CT26 cells with the compounds. Phenformin decreased the oxygen consumption rate (OCR) as expected for a complex I inhibitor. In contrast, oxamate increased OCR. This is also expected because pyruvate would be redirected to mitochondrial oxidative metabolism if LDH is inhibited. Interestingly, OCR was lowest in the phenformin plus oxamate group (Fig. 4B). Methyl succinate can bypass electron transport through complex I because it donates electrons directly to complex II of the mitochondrial electron transport chain. Addition of methyl succinate to phenformin reduced the cytotoxic effect of phenformin (Fig. 4C), again suggesting that complex I inhibition is an important target of the drug.

**Figure 5:**
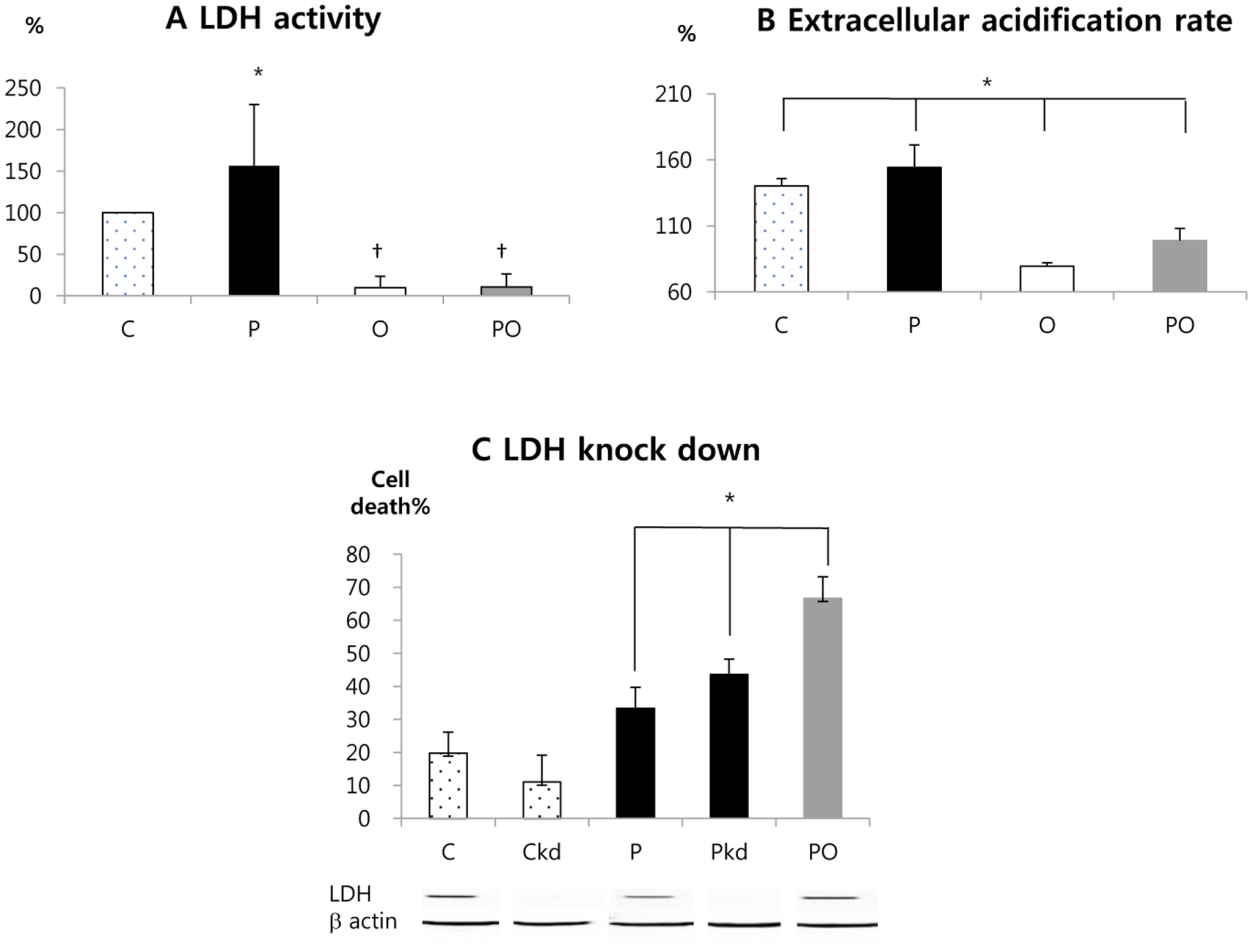
Role of LDH inhibition in enhancing phenformin cytotoxicity. (A) CT26 cells were treated with compounds, as indicated below each bar, for 24 days. Extracts were then prepared for determination of LDH enzyme activity. The Y axis is % of LDH activity when the activity of LDH of the control group is regarded as 100%. (B) Extracellular acidification rate in the presence of the indicated drugs was measured using a Seahorse XF24 instrument as described in Materials and Methods. (C) LDH expression in CT26 cells was repressed using siRNA transfection (groups labelled Ckd and Pkd). A scrambled siRNA transfection was used for groups C, P and PO. The cells were then treated for 24 hours with the indicated drugs and then the number of dead cells in each culture was determined. A western blot for LDHA is shown at the bottom, along with a blot for β-actin as a loading control. C: control, P: phenformin 1 mM, O: oxamate 40 mM, PO: phenformin 1 mM+oxamate 40 Mm, kd: LDH knock down by siRNA. *: P<0.05 compared with the other groups. †: P<0.05 compared with the group C and P.

The direct effects of phenformin and oxamate on LDH activity were also measured. Treatment of cells with phenformin increased LDH activity and treatment with oxamate inhibited LDH activity (Fig. 5A). This is consistent with the known cellular activities of the two drugs. Importantly, oxamate also strongly inhibited LDH activity in phenformin treated cells, indicating that phenformin is not able to reverse the inhibitory effects of oxamate on the enzyme.

Analysis of the extracellular acidification rate (ECAR) using the Seahorse Extracellular Flux Analyzer showed that phenformin increases ECAR, indicating an increase in glycolysis and lactate secretion (Fig. 5B). In contrast, oxamate reduced ECAR, as expected for an LDH inhibitor. Oxamate also strongly inhibited the increase of ECAR resulting from phenformin treatment.

To confirm the importance of LDH inhibition in enhancing the effect of phenformin on cytotoxicity, LDH was knocked down using siRNA transfection. LDH knockdown alone was not cytotoxic to the cancer cells. LDH knockdown increased cancer cell cytotoxicity in the presence of phenformin. However, the siRNA knockdown was less effective than oxamate treatment in enhancing cell death in phenformin treated cells (Fig. 5C). This suggests that knockdown was incomplete or that oxamate has additional targets that influence its ability to work synergistically with phenformin.

### Effect of Phenformin and Oxamate Combination on ROS, ATP, and DNA Damage

Inhibition of complex I is expected to increase superoxide production by mitochondria, enhance formation of other ROS, leading to oxidative stress and potential DNA damage. Inhibition of glycolytic and oxidative metabolic pathways is expected to reduce cellular ATP levels. Such changes may be directly related to the cytotoxicity and synergy of phenformin and oxamate.

**Figure 6:**
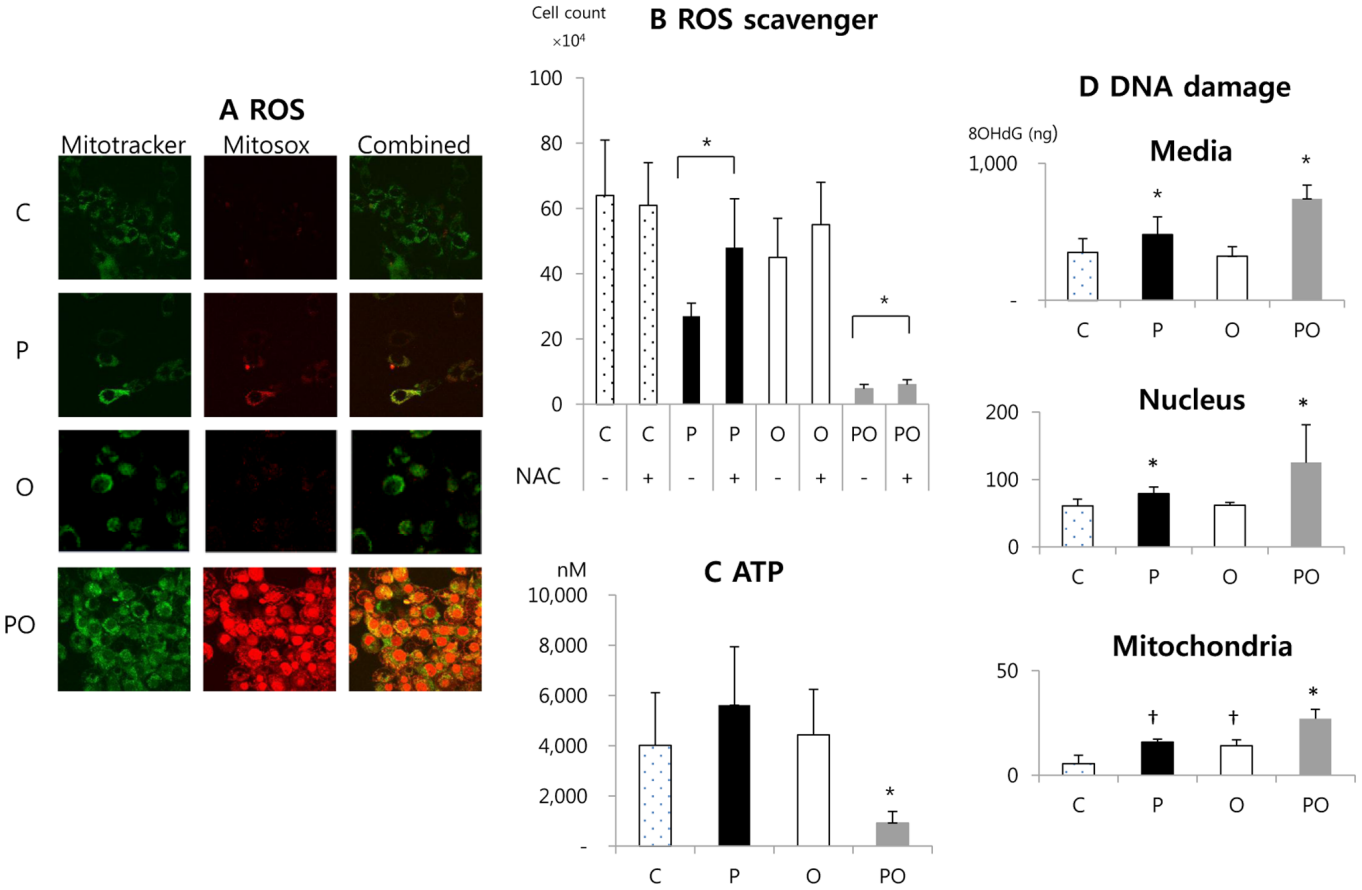
Effects of phenformin and oxamate on ROS, ATP levels, and DNA damage. (A) CT26 cells were treated with compounds as indicated on the left. Eight hours after drug treatment MitoSOX staining was used to examine cellular levels of superoxide by confocal imaging. Mitotracker Green was used to label mitochondria. Magnification 100X. (B) CT26 cells were treated with the indicated compounds in the presence or absence of the ROS scavenger NAC (N-acetyl-cysteine). NAC was added to cultures 6 hours prior to adding phenformin or oxamate. Live cell number was then determined 24 hours after drug treatment. (C) Cells were treated with phenformin, oxamate, or both for 24 hours and then cellular ATP levels were measured. (D) Cells were treated as indicated for 24 hours and then the medium was collected and the cells fractionated into nuclear and mitochondria enriched fractions. In each compartment the level of oxidative damage to DNA was estimated using an ELISA to detect 8-OHdG. C: control, P: phenformin 1 mM, O: oxamate 40 mM, PO: phenformin 1 mM+oxamate 40 mM. *: P<0.05 compared with the other groups. †: P<0.05 compared with the group C and PO.

As indicated by MitoSox Red staining, phenformin induced elevated production of mitochondrial superoxide (Fig. 6A). Oxamate alone did not affect mitochondrial ROS production. However, the addition of oxamate with phenformin greatly potentiated ROS production. NAC is a ROS scavenger that is known to reduce cellular oxidative stress. NAC treatment reduced cell death in phenformin treated cells (Fig. 6B). NAC also reduced cell death in the phenformin plus oxamate treated cells, but was much less effective in this group.

Phenformin and oxamate single treatment tended to increase ATP production compared to the control (no statistical differences) (Fig. 6C). However, addition of oxamate plus phenformin greatly decreased ATP levels compared to untreated cells, suggesting a synergistic effect.

As a measure of oxidative DNA damage, 8-OHdG in the culture medium, nuclei, or mitochondria was measured. In all three compartments, the phenformin treatment group showed increased DNA damage compared to the control group (Fig. 6D). Oxamate alone showed increased DNA damage in mitochondria compared with the control, when added together with phenformin DNA damage was significantly increased.

### Death of Cancer Cells occurs through both Apoptotic and PARP-dependent Pathways

**Figure 7:**
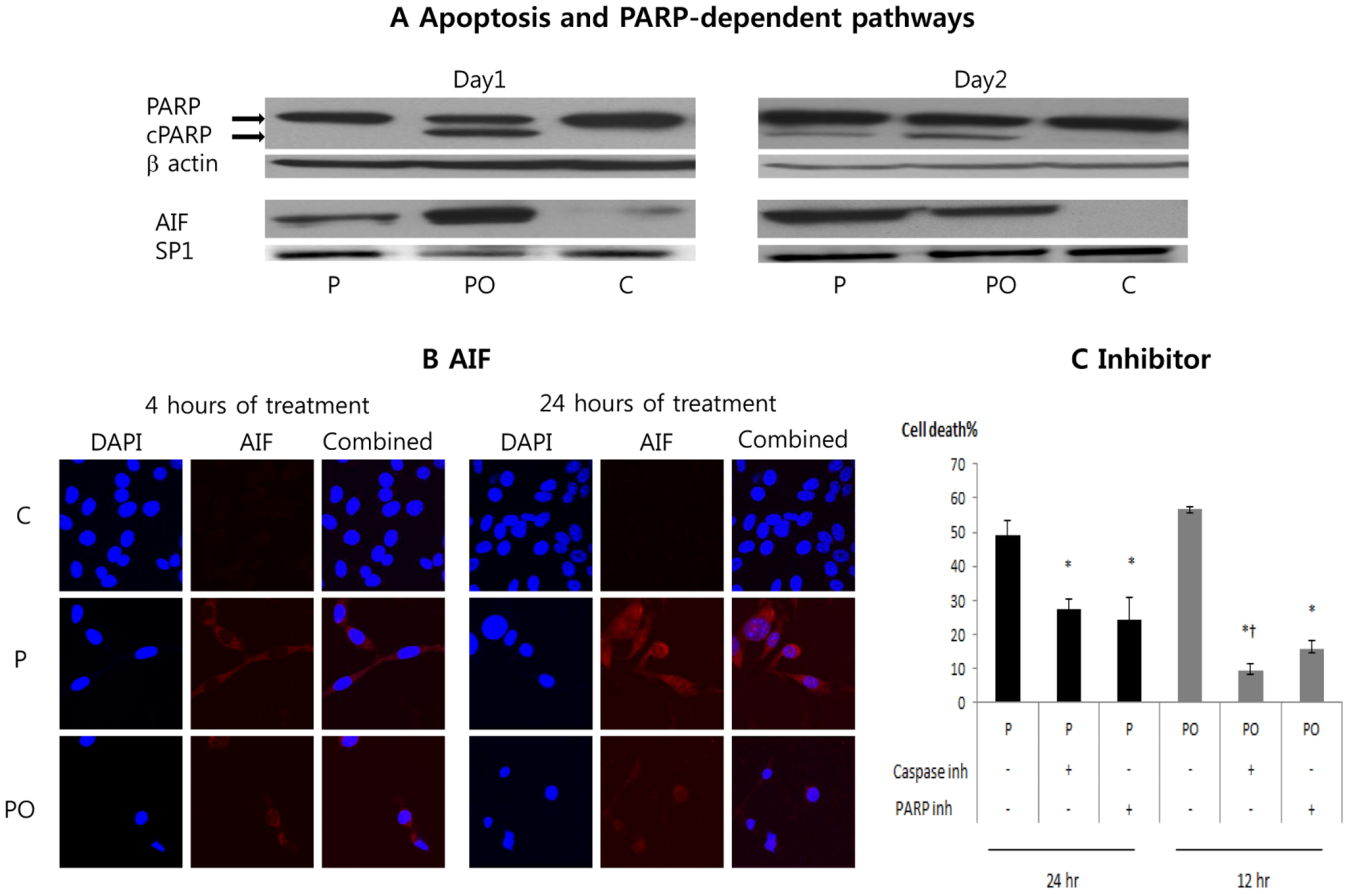
Cell death pathways induced by phenformin and oxamate. (A) CT26 cells were treated as indicated at the bottom of each lane. Experiments were performed after either 1 day (left) or 2 days (right) of treatment. Western blot analysis of cPARP in total cell extracts was used as an estimate of apoptotic cell death. Western blot analysis of nuclear AIF was used as an estimate of PARP-dependent cell death. β-actin and SP1 were used for protein loading controls. (B) AIF (red) was detected by immunofluorescence in cells that had been treated with the compounds indicated on the left and for the time indicated at the top. DAPI was use to stain nuclei (blue). (C) Cells were treated with phenformin or phenformin plus oxamate in the presence or absence of either a pan-caspase inhibitor or a PARP inhibitor. The percentage of dead cells was determined 24 hours after treatment in the P group and 12 hours after treatment in the PO group. C: control, P: phenformin 1 mM, PO: phenformin 1 mM+oxamate 40 mM. *: p<0.05 compared with the control group. †: P<0.05 compared with PO+PARP inhibitor.

We have previously found that the biguanide metformin kills breast cancer cells through both apoptotic and PARP-dependent pathways [22]. We therefore examined cell death in phenformin and oxamate treated cells in more detail. Cell death was more rapid in the phenformin plus oxamate group than in the phenformin alone group (Fig. 7). In both groups, hallmarks of both apoptosis and PARP-dependent pathways were detected. Cleaved PARP (cPARP) is a hallmark of caspase-dependent apoptosis. In western blot analysis, cPARP was induced on day 1 in the group phenformin plus oxamate group and on day 2 in the phenformin alone group (PO, Fig. 7A). Release of Apoptosis Inducing Factor (AIF) from mitochondria followed by nuclear uptake of the protein is a hallmark of PARP-dependent cell death [27]. After 1 day treatment, the level of AIF in nuclei was higher in the PO group than in the P group. After 2 day treatment, AIF in the nucleus started to decrease in the PO group but increased in the P group. Both cPARP and nuclear AIF were almost undetectable in the control group (Fig. 7A).

By confocal microscopy, AIF was evident in the nuclei by 4 hours after drug treatment in the PO group, and at 24 hours after treatment with phenformin alone (Fig. 7B). In the PO group, cells were largely disrupted by 24 hours and the AIF signal was very faint. AIF was almost undetectable at both time points in the control group.

To further demonstrate the involvement of both caspase and PARP-dependent cell death mechanisms, cells were treated with phenformin or phenformin plus oxamate in the presence and absence caspase or PARP inhibitors. Cell death induced by phenformin or phenformin plus oxamate was significantly reduced by treatment with either a pan-caspase inhibitor or a PARP inhibitor (Fig. 7C).

### Effects of Phenformin and Oxamate on Tumors *in vivo*

#### Tumor size

**Figure 8:**
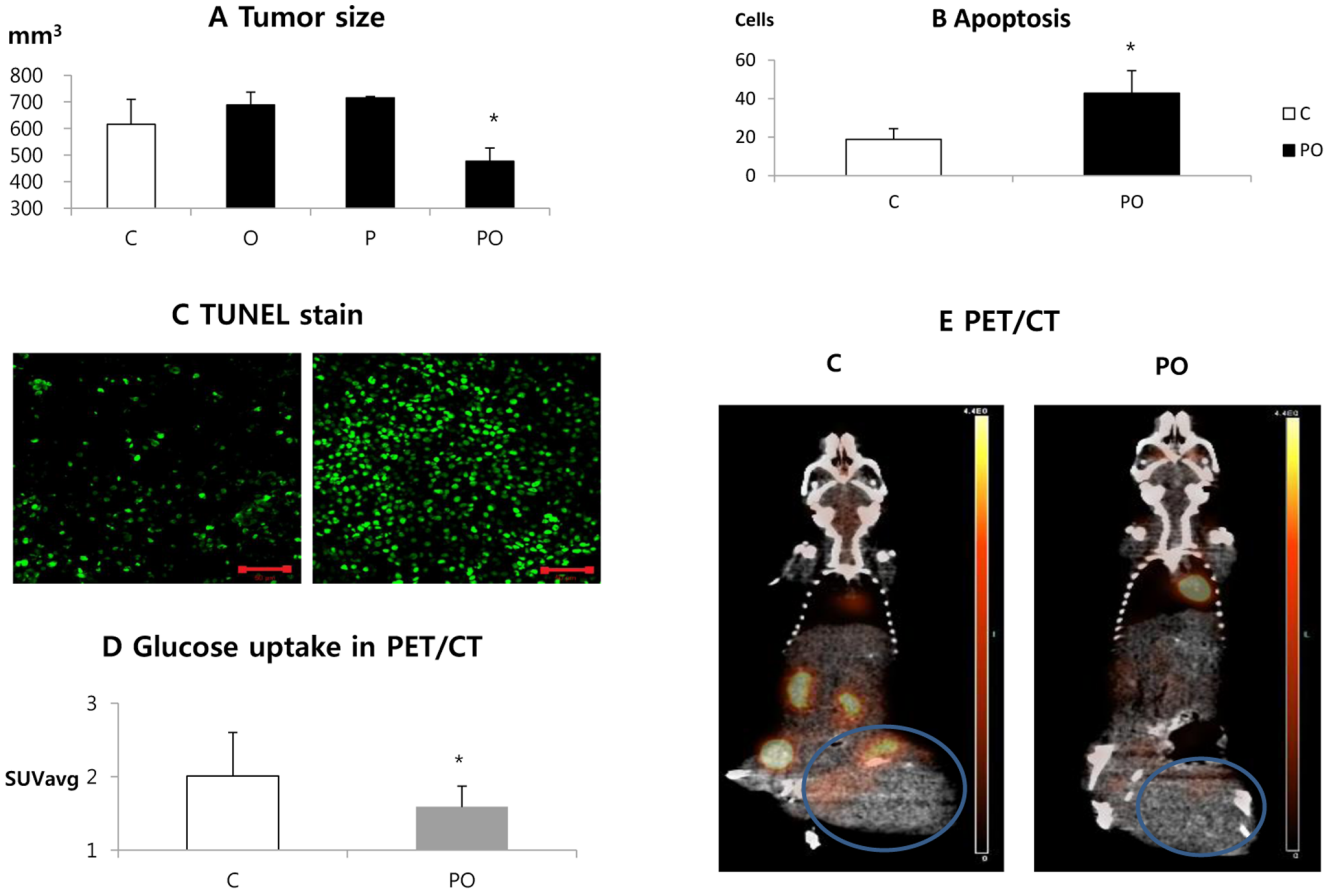
Effects of phenformin and oxamate on tumors *in vivo.* (A) CT26 tumors were developed in syngeneic host mice. Three days after cell injection the mice were treated with oxamate, phenformin, or both daily for 21 days. Average tumor size for each group on day 21 of treatment is shown. Group PO tumors were significantly smaller compared to the other groups (P<0.05). There was no significant difference in tumor sizes between groups C, O, and P. (B, C) Tumor samples were processed to examine TUNEL positive cells as a measure of apoptosis. Cells which showed strong TUNEL positive were counted in three sections (304 µm×304 µm) in each mouse at 20X by confocal microscopy. The PO group showed significantly higher apoptosis than group C (apoptotic cells: 42.8±23.5 vs. 18.9±11.1) (P = 0.001). (D, E) Tumor bearing mice were subjected to PET/CT scanning to determine the effect of phenformin plus oxamate on glucose uptake. Group C showed significantly higher glucose uptake compared to the PO group (SUVavg: 2.0±0.6 vs. 1.6±0.3) (P = 0.033).

Tumors were developed from CT26 colon cancer cells in syngeneic immune-competent host mice as described in Materials and Methods. Three days after injection of tumor cells, treatment with phenformin, oxamate, or both was initiated. Treatment was performed every day for 21 days and tumor sizes at 21 days are shown in Fig. 8A. The control group and the groups treated with either oxamate or phenformin alone had tumors that were not significantly different in size. However, the group treated with a combination of phenformin plus oxamate had substantially smaller tumors than the other groups. Mean tumor size was 616±94 mm^3^ in the control group, 731±31 mm^3^ in the P group, 769±1084 mm^3^ in the O group, and 476±50 mm^3^ in the PO group (PO vs. other groups, P<0.05). Thus the combination of phenformin and oxamate is effective in slowing CT26 tumor growth.

#### Effect of phenformin and oxamate on tumor apoptosis

End stage tumors were harvested from the control and PO treatment groups described above. The tumors were then processed to examine TUNEL positive cells as an indicator of apoptotic cell death (Fig. 8B). Representative TUNEL staining in each group is shown in Fig. 8C. The PO group showed significantly higher levels of apoptosis than the control group (Fig. 8B) (apoptotic cells 42.8±23.5 vs. 18.9±11.1 in the 304 µm×304 µm section) (P = 0.001). Thus enhanced apoptosis corresponds to reduced tumor growth following treatment with phenformin and oxamate.

#### ^18^F-FDG small animal PET/CT

Reprogramming of cancer cells allows them to take up high levels of glucose and process it through glycolysis. This characteristic of tumors allows them to be imaged by PET scanning using a radioactive glucose analogue that is non-metabolizable (^18^F-FDG). PET scanning is thus an indicator of glucose uptake and metabolic activity of the tumor cells. Mice carrying CT26 tumors were subjected to PET/CT scanning following 21 days of treatment (Fig. 8D, 8E). Glucose uptake (SUV_avg_) of tumors in the untreated control group was significantly higher than that in the phenformin plus oxamate treated group (2.0±0.6 vs. 1.6±0.3; P = 0.033). Representative PET/CT images of each group are shown in Fig. 8E. Thus the combination of phenformin plus oxamate is able to significantly alter tumor metabolism and this correlates with reduced tumor growth.

## Discussion

In this study, we evaluated whether phenformin has a greater anti-cancer cell effect than metformin and investigated the effects of combining oxamate with phenformin on cancer cells. Furthermore, we elucidated important aspect of the mechanisms and pathways in cancer cells that are altered by these two drugs. Our results suggest that phenformin has higher cytotoxicity and growth suppression towards cancer cells than metformin. Moreover, addition of oxamate not only reduces lactic acid production but also enhances the anti-cancer effect of phenformin. Our data suggest that this synergistic anti-cancer activity involves simultaneous inhibition of complex I and LDH by phenformin and oxamate, respectively.

The EC_50_ of metformin for inducing cancer cell death was 840 times higher than that of phenformin in E6E7Ras cells, a model of HPV+ head and neck cancer. Phenformin was also more potent than metformin in various other cancer cell lines, including those from melanoma, breast, colon, prostate, and lung cancer. One previous study similarly showed that phenformin is more cytotoxic than metformin [24]. In our study, the cytotoxic effect of phenformin was related to inhibition of complex I of the electron transport chain. Metformin is also known to be taken up and concentrated in mitochondria, where it inhibits complex I [12], [13]. A possible reason for the stronger potency of phenformin is that phenformin is more lipid-soluble than metformin and therefore crosses mitochondrial membranes more easily [28] and inhibits complex I much more rapidly [12], [24].

In our experiments, phenformin inhibited complex I. Methyl succinate, which bypasses complex I by donating electrons directly to complex II of the mitochondrial electron transport chain, reduced cytotoxic effects of phenformin. This suggests that inhibition of complex I is responsible for phenformin's anti-cancer effects. However, the reversal was not complete, implying phenformin may act through multiple pathways [7]–[11].

Phenformin increased mitochondrial ROS production. Inhibition of complex I increases the aberrant flow of electrons to oxygen and creates superoxide within the mitochondrial matrix. Superoxide radical anion leakage from the electron respiratory chain causes damage to mitochondrial proteins, lipids, and nucleic acids [24]. While normal cells can effectively produce a mitochondrial form of superoxide dismutase (MnSOD) to detoxify ROS, many cancer cells have low expression levels of MnSOD [29] and cannot effectively detoxify ROS. Therefore, cancer cells can be more vulnerable to overproduction of ROS. Cancer cell lines that express higher levels of MnSOD were more resistant to metformin cytotoxicity [22], [30], indicating that biguanide anti-cancer action could be closely related to ROS production. Treatment with the ROS scavenger NAC significantly reduced the anti-cancer effect of phenformin in this study, confirming the importance of ROS in mediating cell death.

In our study, oxamate alone was poorly effective in killing cancer cells, consistent with a previous study [31]. However, oxamate greatly enhanced the cytotoxic effect of phenformin in a dose- and time-dependent manner. There are no previous reports on cancer cell cytotoxicity of the combination of phenformin and oxamate.

Three mechanisms of this synergy by oxamate are proposed here: reversal of the lactic microenvironment, increased mitochondrial respiration (OXPHOS) and production of ROS, and depletion of ATP.

First, elevation of LDH activity has been well documented in a variety of human cancer cell lines and tissue sections and LDH overexpression is a negative prognostic marker in various cancers [32]. LDH catalyzes conversion of pyruvate into lactate to ensure a rapid and constant supply of ATP. The produced lactate is transported out of the cell and results in elevated lactate and reduces pH in the tumor microenvironment. High tumor microenvironmental lactate is related to cancer cell metastasis, impaired host immune response, and poor prognosis of cancer [14], [15].

Phenformin treatment accelerated LDH activity and lactate production in this study (Fig. 3B). Impairment of complex I by phenformin leads to impairment of the oxidative phosphorylation pathway, and promotes the glycolytic pathway with compensatory acceleration of LDH activity [24]. Oxamate inhibited LDH activity and prevented lactate production and the pH decrease promoted by phenformin. Oxamate even reversed the acidic environment of cancer cells: the pH of the culture medium on the third day of treatment was 6.5 in the control group C, 6.2 in the P group, and 7.4 in the PO group. Seahorse XF24 extracellular flux analysis experiments showed that phenformin increases extracellular acidification rate (ECAR) which means phenformin accelerates glycolysis and lactate secretion. Oxamate reduced ECAR, and addition of oxamate to phenformin inhibited the increase of ECAR by phenformin.

Second, oxamate increases total mitochondrial respiration through LDH inhibition [16]. Our experiments also showed oxamate monotherapy increases oxygen consumption rate (OCR, mitochondrial respiration). Activity of complex I and LDH are closely connected and compete through the mitochondrial NADH/NAD^+^ shuttle systems [33]. LDH requires NADH in the cytoplasm during glycolysis whereas complex I requires NADH for electron transfer in the mitochondria. This competition for NADH is most likely at the core of the slowdown of mitochondrial respiration in cancer cells [33]. Oxamate shifts this balance towards dominance of mitochondrial respiration by blocking LDH. A shift toward mitochondrial respiration will increase ROS production, especially when complex I activity is impaired by phenformin. We suggest that, in the presence of phenformin, addition of oxamate greatly increases mitochondrial ROS production due to increased aberrant flow of electrons to oxygen through complex I. This causes mitochondrial damage and disruption of the organelle, leading to general cellular oxidative stress, and oxidative damage of nuclear DNA. This is supported by the data in Figures 6A and 6D which show that MitoSOX stains both mitochondria and nuclei and that there is oxidative damage of DNA in both compartments. MitoSOX is a selective indicator of mitochondrial ROS production and normally stains mitochondrial DNA. Excessive nuclear staining with MitoSOX indicates damaged mitochondrial membranes and nuclear uptake of the mitochondrial-derived oxidized MitoSOX. The production of ROS was so extensive that the ROS scavenger, NAC, could not effectively reduce cell death in the phenformin plus oxamate group.

Third, the energy demand of cancer cells is high to support biosynthetic reactions required for proliferation. Therefore, tumor cells do not adapt efficiently to metabolic stress and can be induced to die by metabolic catastrophe [34]. Phenformin single agent treatment tended to increase ATP production (no statistical significance). Biguanides increase glucose uptake and accelerate glycolysis due to mitochondrial impairment [24], [34]. Increased glucose uptake and glycolysis maybe the reason why ATP production is increased in phenformin treated cells. Phenformin plus oxamate greatly decreased ATP production (Fig. 6C) and this correlates with synergistic killing of cancer cells by the two drugs. In a recent report, a combination of metformin and the glycolysis inhibitor 2-deoxyglucose (2DG) showed a synergistic effect on various cancer cell lines and inhibited tumor growth in a mouse xenograft model in association with a decrease in cellular ATP [35]. 2DG is a glucose molecule which has the 2-hydroxyl group replaced by hydrogen, so that it cannot undergo further glycolysis. Combined incubation of 2-DG with phenformin showed greater growth inhibitory effects than metformin with 2-DG in *in-vitro* studies [36]. These reports, together with the data presented here, indicate that coupling biguanides with compounds that inhibit glycolysis is an effective means of killing cancer cells.

To further investigate the effect of LDH inhibition, we examined the effects of oxamate and siRNA-mediated LDH knockdown on cancer cell death. LDHA is commonly overexpressed in cancer cells [37] therefore only the LDHA gene product was targeted for knockdown in this study. In the untreated control group, LDH knockdown did not increase cancer cell cytotoxicity. In contrast, LDH knock down increased cancer cell cytotoxicity in phenformin treated cells. As compared to phenformin plus oxamate, phenformin plus LDH knockdown had a weaker cytotoxic effect. This suggests LDH knockdown was incomplete or that oxamate may have other effects in addition to LDH inhibition (Fig. 5C). Thornburg et al. [38] demonstrated that oxamate also inhibits aspartate aminotransferase (AAT). Oxamate is a more potent inhibitor of LDHA than AAT, but inhibition of both enzymes could contribute to the effects of oxamate in the presence of phenformin [38]–[40]. As part of the malate-aspartate shuttle, AAT is required to shuttle electrons from glycolysis-derived cytoplasmic NADH to mitochondrial NADH, which can transfer electrons to Complex I for oxidative phosphorylation. In this scenario, we would expect oxamate inhibition of AAT to reduce the toxicity of phenformin because fewer electrons would flow through Complex I. Other enzymes such as hexokinase [40], pyruvate carboxylase, and pyruvate translocator [41] have also been suggested as targets of oxamate. These additional targets of oxamate could explain why the phenformin plus oxamate combination was more effective than phenformin combined with LDH knockdown.

Cancer cells died through apoptosis and PARP-dependent pathways in both the P and PO groups. ROS are known to be involved in both death mechanisms [42], [43]. Apoptosis, a form of programmed cell death, is a caspase-dependent cell death [44] and cleaved PARP (cPARP) is a hallmark of caspase-dependent apoptosis. PARP-dependent cell death is a unique form of programmed cell death involving PARP-1 activation, PAR polymer formation, translocation of apoptosis inducing factor (AIF) from mitochondria to the nucleus, and AIF-mediated chromatin condensation/large scale DNA fragmentation [45]. We showed translocation of AIF into the nuclei in the P and PO groups, a hallmark of PARP-dependent cell death. Cell death was reduced by treatment with pan-caspase inhibitor or PARP inhibitor. In total, our results indicate that phenformin or phenformin plus oxamate kill cancer cells through two pathways as previously shown for metformin in breast cancer cells [22].

We also examined the effects of these compounds on CT26 tumors *in vivo*. In this study, there were no differences in tumor sizes between the control group and the groups treated with oxamate or phenformin alone (Fig. 8A). In contrast, phenformin plus oxamate reduced tumor growth in mice. Thus the effects of the combination are similar *in vivo* and in cell culture.

Recently two *in vivo* studies using phenformin single agent treatment were published. One study reported that phenformin showed significant growth inhibition of breast cancer xenografts in mice [6]. The other reported that phenformin treatment caused increased survival and slower lung cancer progression in mice with *Kras* and *Lkb1* mutation, suggesting phenformin as a cancer metabolism-based therapeutic [46].

Other studies using oxamate single agent treatment in tumor-bearing animals have also been performed. These have shown divergent results. In agreement with our results, Yaromina et al. [47] showed no effect of oxamate in nude mice implanted with human colorectal adenocarcinoma WiDr. In contrast, Thornburg et al. [38] found tumor size reduction with oxamate treatment of MDA-MB-231 breast tumors in athymic mice.

Our experiments used mouse colon cancer cells implanted in syngeneic immune-competent mice. There are a number of possible reasons for the differential results obtained by various groups for the effects of these compounds on tumor growth *in vivo.* First, cytotoxicity *in vitro* may not reflect tumor reduction effects *in vivo* [47]. Second, phenformin’s anti-cancer potency is different among various cell lines. For example, the CT26 line we used was more resistant than other cell lines to phenformin single agent treatment in cell culture studies. Third, activation of alternative pathways such as glutaminolysis may contribute to contradictory results in *in vivo* experiments. Inhibition of a single enzyme may not be sufficient and several regulators of metabolism could need to be inhibited simultaneously to achieve significant results [47]. Fourth, all studies except ours used immune-deficient mice. Immune responses in immune-competent mice may influence the effects of the compounds on tumor growth. Phenformin and oxamate are expected to alter lactate in the tumor microenvironment in opposite directions. Altered lactate in the tumor microenvironment may have influenced host immune responses against cancer cells in these experiments. Lactate in the tumor microenvironment has previously been shown to affect immune responses [48]–[51] and to affect responses of tumors to therapy [14], [15].

Another point worth mentioning is that the number of apoptotic cells in tumor sections was relatively small (apoptotic cells PO 42.8±23.5 vs. C 18.9±11.1 in the 304 µm×304 µm section). This is in line with previous reports. MCF7 and MDAMB231 tumors treated with phenformin showed few apoptotic cells but significant suppression of the number of mitotic cells [6]. This may indicate that tumor growth inhibition was the result of reduced proliferation rather than increased cell death in *in vivo* environments. In our experiments, phenformin plus oxamate showed decreased glucose uptake compared to the control in PET/CT. Decreased signal in PET/CT is a surrogate marker of decreased glucose utilization and proliferation of cancer [52]. This is consistent with the observed effects of combined phenformin and oxamate on tumor cell metabolism in culture and suggests that the drugs promote similar metabolic changes in tumors *in vivo*.

Repurposing phenformin and oxamate as anti-cancer drugs would be cost effective and they are relatively safe drugs compared with existing chemotherapeutic agents. Despite the higher rate of lactic acidosis, phenformin is still legally prescribed in Italy, Brazil, Uruguay, China, Poland, Greece and Portugal. Renal failure patients might show increased toxicity by phenformin treatment due to decreased excretion [53]. Oxamate is not an FDA approved drug but as a structural analog of pyruvate it is known to be relatively safe. Individuals with hereditary LDHA deficiency show myoglobinuria only after intense anaerobic exercise (exertional myoglobinuria) but do not show any symptoms under ordinary circumstances [54]. Therefore, we can easily and safely apply these agents in clinical practice as single agents or as adjuvants to existing chemotherapeutic agents.

**Figure 9:**
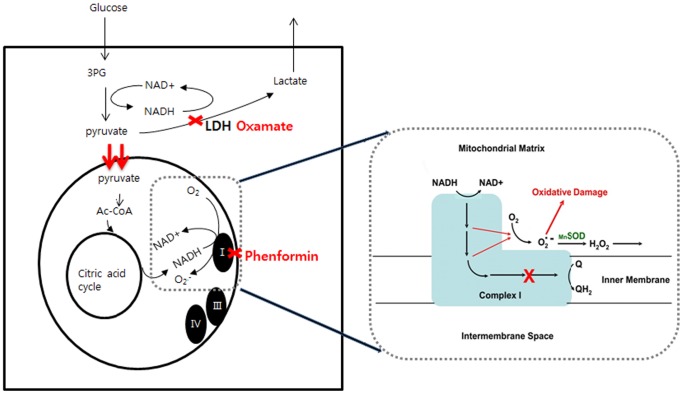
Model of phenformin and oxamate activity in tumor cells. We propose that the two drugs act synergistically by simultaneous inhibition of complex I and LDH. Phenformin increases ROS production by inhibiting mitochondria complex I. Inhibition of LDH by oxamate results in decreased ATP levels and elevated ROS production in the presence of phenformin because of increased flow of electrons through complex I.

Based on the unique cancer metabolism and mechanism of action of these two drugs, our working model for the mechanism of phenformin and oxamate is as follows: The cytotoxic effects of phenformin are related to inhibition of complex I of the mitochondrial respiratory chain. Inhibition of complex I increases electron transport to O_2_ and results in over production of ROS within the mitochondrial matrix that causes damage to mitochondrial DNA, proteins, and membranes. This eventually leads to general cellular oxidative damage and cell death. Inhibition of LDH by oxamate results in improvement of the acidic cancer microenvironment and a decrease in ATP production. An increase in mitochondrial respiration induced by oxamate leads to increased ROS production and DNA damage in the presence of phenformin, leading to rapid apoptosis and PARP-dependent cancer cell death (Fig. 9).

For future studies, the effects of oxamate other than LDH inhibition should be investigated. It would be interesting to know whether cancer cells with different levels of MnSOD show different sensitivity to phenformin and oxamate treatment. Finally, clinical investigations with these drugs are required.

## Conclusion

Phenformin is more cytotoxic towards cancer cells than metformin. Phenformin and oxamate have synergistic anti-cancer effects by simultaneous inhibition of complex I in the mitochondria and LDH in cytosol, respectively.
